# Susceptibility to first choice antimicrobial treatment for urinary tract infections to E*scherichia coli* isolates from women urine samples in community South Brazil

**DOI:** 10.1016/j.bjid.2022.102366

**Published:** 2022-05-18

**Authors:** Zuleica Naomi Tano, Renata K. Kobayashi, Evelyn Poliana Candido, Juliana Buck Dias, Luis Felipe Perugini, Eliana Carolina Vespero, Wander Rogerio Pavanelli

**Affiliations:** a Universidade Estadual de Londrina, Departamento de Medicina Interna, Londrina, PR, Brazil; b Universidade Estadual de Londrina, Departamento de Microbiologia, Londrina, PR, Brazil; c Universidade Estadual de Londrina, Departamento de Patologia, Análises Clínicas e Toxicológicas, Londrina, PR, Brazil; d Universidade Estadual de Londrina, Centro de Ciências Biológicas, Departamento de Ciências Biológicas, Laboratório de Imunoparasitologia de Doenças Negligenciadas e Câncer (LIDNC), Londrina, PR, Brazil

**Keywords:** Urinary tract infection, Bacteriuria, Drug resistance, Uropathogenic, Escherichia coli

## Abstract

*E. coli* is the main pathogen of UTI. It is important to be aware the local epidemiological data for an appropriate initial treatment. Resistance to antimicrobial agents has increased, especially to first-choice antibiotics in the treatment of cystitis. There are few studies on the sensivity profile of community uropathogen in our region.

**Objective:**

To characterize antimicrobials the sensitivity profile to E. coli isolated from urocultures of women treated at Basic Health Units and Emergency Care Units of Londrina- Paraná- Brazil during a period of 12 months (June 1, 2016 to June 1, 2017).

**Methodology:**

A cross-sectional study was carried out from June 2016 to June 2017. All urine samples collected in the Basic Health Units and Emergency Departments in the city of Londrina (Paraná State, Brazil) were sent to a Central Laboratory where the identification and antimicrobial susceptibility testing were performed. Clinical Laboratory Standards Institute (CLSI) breakpoints were used for the interpretation of susceptibility testing results.

**Results:**

56,555 urine cultures were performed in the period, of which 8,832 were positive, of which 5,377 were women. Of these samples, 4.7% were enterobacteria producing extended-spectrum beta-lactamases (ESBL) and 15.5% resistant to quinolones. TMP- SMX was resistant in more than 30% of the samples in all age groups. Among quinolone-resistant isolates, resistance to cephalothin, ampicillin and sulfamethoxazole-trimethoprim was greater than 60%. Nitrofurantoin was the only antimicrobial that showed 90% of sensitivity.

**Conclusion:**

The antimicrobials sensitivity profile was similar to that reported in the literature, with TMP- SMX resistance greater than 30% in the studied samples. Nitrofurantoin maintains high sensitivity rates greater than 90%. Resistance to quinolones increases proportionally with age, as well ESBL.

## Background

Urinary Tract Infection (UTI) is the most common outpatient infection, and the second most frequent after respiratory tract infection.^1^ Women are more affected than men due to the shorter distance between the female urethra and bladder, which makes bacterial colonizers ascend to kidneys before they are removed by micturition.^2^ Symptomatic infection is more frequent in women aged 15-29 years (12.6%), whereas the incidence in men comprises 3% in USA.^3^

Among healthy women aged 18-39 years, 80% of UTIs are caused by *E. coli*, which is the target of empirical therapy. However, significant variations in antimicrobial susceptibility have been observed in several countries over the last years, with the progressive emergence of resistance to fluoroquinolones and other antibiotics commonly used for empirical treatment of community-acquired UTIs. Presence of extended spectrum beta-lactamases (ESBLs) in Latin America increased from 1.7% to 7.1 – 12.5%.^4^, ^5^, ^6^

Since 2011, the Infectious Diseases Society of America (IDSA) has recommended that trimethoprim-sulfamethoxazole (cotrimoxazole), nitrofurantoin, fosfomycin, or pivmecillinam, should be used whether local resistance rates of uropathogens causing acute uncomplicated UTIs do not exceed 20%, or whether the infecting strain is known to be susceptible to these drugs.^7^ Currently, guidelines recommend fosfomycin trometamol and nitrofurantoin as the first-choice treatment for patients with uncomplicated UTIs, for which cotrimoxazole is the third option.^5^ In Korea, nitrofurantoin, fosfomycin and pivmecillinam are the treatment of first choice for uncomplicated UTIs, whereas cotrimoxazole may be used only when antimicrobial susceptibility testing confirms drug sensitivity.^8^

The appropriate choice of antibiotics in patients with suspected uncomplicated UTI should be based on up-to-date surveillance data from patients in primary care settings. Thus, prospective surveillance of antibiotic resistance patterns in uropathogens from all patients attending these settings is crucial for guiding first- and second-line antibiotic selection.^4^

This study aimed to evaluate the antimicrobial susceptibility profile for first-line treatment for UTI caused by *E.coli* isolated in urine samples of women in the community and presence of extended-spectrum beta-lactamase (ESBL).

## Material and methods

A cross-sectional study was carried out from June 2016 to June 2017. All urine samples collected at the Basic Health Units and Emergency Departments in the city of Londrina (Paraná State, Brazil) were sent to a Central Laboratory where the identification and antimicrobial susceptibility testing were performed. First-morning midstream urine samples were collected, of which 10 microliters were inoculated onto chromogenic media CPS ID 3 (BioMérieux, Marcy I’Étoile, France), and incubated overnight at 36°C. Urine culture was considered positive according to the following criteria: growth of a single bacterium (pure culture) and colony counts > 10^5^ colony-forming units (CFU)/mL. Bacteria were identified according to phenotypic characteristics displayed on CPS ID 3 medium or by using the Vitek® 2 automated system (BioMérieux, Marcy I’Étoile, France). Urine culture of men and uropathogens other than *E. coli* were excluded. Data such as age and pregnancy status were analyzed through the WebSaúde system of Londrina city.

### Antimicrobial susceptibility testing (AST)

AST was performed by using the AST-238 card, whose results were evaluated with the VITEK® 2 (BioMérieux, Marcy-I'Etoile, France) system. The following antibiotics were tested: amikacin (AST-N054 only), ampicillin, amoxicillin/clavulanic acid, aztreonam (AST-N054 only), cefalexin, cefepime, cefotaxime, ceftazidime, cefoxitin, cefuroxime, ciprofloxacin, norfloxacin, ertapenem, gentamicin, meropenem, nalidixic acid, nitrofurantoin, piperacillin, piperacillin/tazobactam, and trimethoprim. Clinical Laboratory Standards Institute (CLSI) breakpoints were used for the interpretation of susceptibility testing results. Isolates were classified as susceptible (S), intermediately resistant (I) or resistant (R) to the aforementioned antimicrobials, respectively, according to the following MIC breakpoints (µg/mL): ampicillin, ≤ 8, 16, ≥ 32; amoxicillin-clavulanate, ≤ 8/4, 16/8, ≥ 32/16; cefuroxime axetil, ≤ 4, 8-16, ≥ 32; norfloxacin, ≤ 4, 8, ≥ 16; ciprofloxacin, ≤ 1, 2, ≥ 4; cotrimoxazole, ≤ 2/38, ≥ 4/76; nitrofurantoin, ≤ 32, 64, ≥ 128; fosfomycin was evaluated by disk diffusion method (Oxoid, Cambridge, UK). The isolates were screened for ESBL production through chromID® ESBL agar plate test (BioMérieux, Marcy l’Étoile, France).

### Statistical analysis

The results were stored and analyzed using SPSS 17. The participants were subdivided into four age groups (< 15, 15-45, 46- 59, and > 60 years), with their respective bacterial isolates. Two-sided chi-square test and Fisher's exact test were used to assess whether there were differences regarding the antimicrobial resistance profile of *E. coli* isolates across age groups. Significant differences in the prevalence of antimicrobial resistance between age groups were determined by odds ratio with 95% confidence intervals and *p*-value < 0.05. The study was approved by Ethics and Research Committee of the State University of Londrina (CAAE 56869816.0.0000.5231) and authorized by the Health Department of Londrina, Paraná.

## Results

**Fig. 1:**
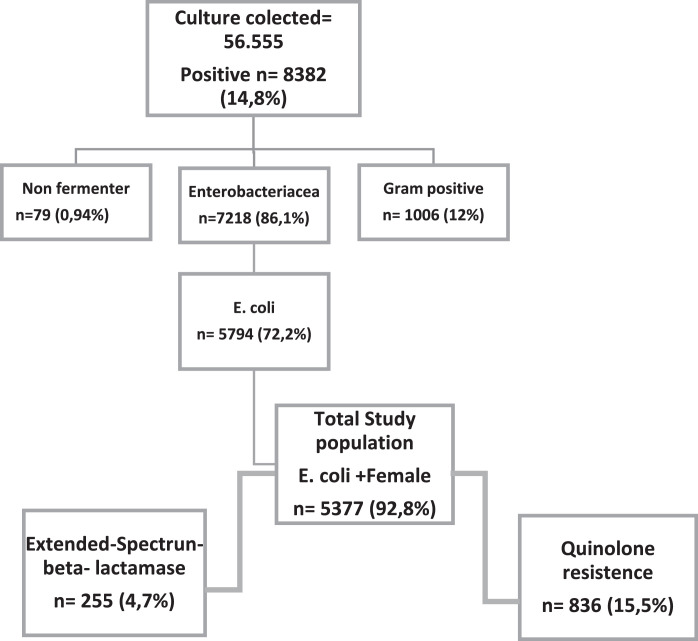
Study population.

A total of 56,555 urine cultures were performed, of which 8,382 were positive, and out of these 5,794 (72.2%) were positive for *E. coli.* Women accounted for 92.8% (5,377/5,794) positive cultures. Moreover, 10% of these women were pregnant, as shown in Fig. 1. ESBL production was detected in 4.7% (*n* = 255) of the isolates.

The average age of women was 47 years (ranging from 0 to 101 years). In this study, 1,777 (33%) women were aged 60 years and over had isolates presented with a higher frequency of ESBL production (8.3%) when compared to other age groups. Susceptibility rates to the quinolone nalidixic acid and the fluoroquinolones ciprofloxacin and norfloxacin were 73.3%, 85.7%, and 85.9%, respectively. For the isolates resistant to these three antimicrobials, susceptibility to fosfomycin was 98.3%.

**Fig. 2:**
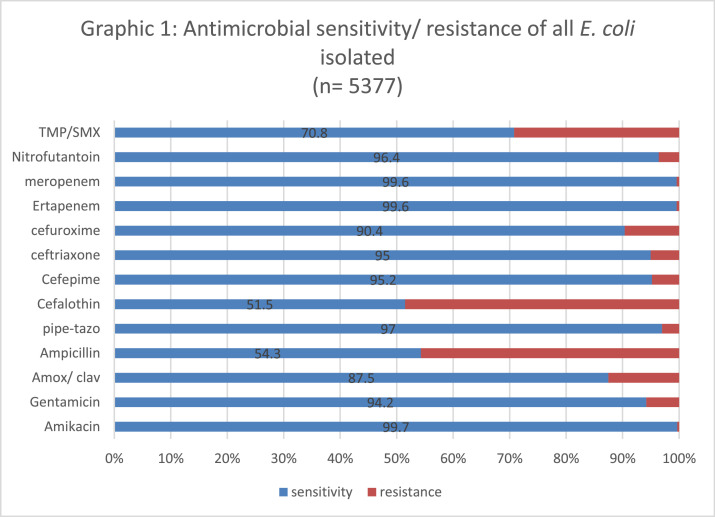
Antimicrobial sensitivity/ resistance of all E. coli isolated (*n* = 5377).

The lowest susceptibility rate was observed for cephalothin (51.8%), followed by ampicillin (54%); while amikacin, ertapenem and meropenem presented the highest susceptibility rate (99.7%). Fig. 2 shows the sensitivity and resistance of all 4,377 samples.

### Susceptibility to first-line UTI antimicrobials agents according to age

Cephalotin (48.1%) and ampicillin (52%) presented the lowest susceptibility patterns, regardless of age. Trimethoprim-sulfamethoxazole (TMP-SMX) displayed resistance rates greater than 30% in all age groups, whereas cefuroxime presented a susceptibility rate greater than 90%. Susceptibility to gentamicin, amoxicillin/clavulanic acid, piperacillin-tazobactan, cephalothin, cefepime, ceftriaxone, cefuroxime, nalidixic acid, norfloxacin, ciprofloxacin and TMP-SMX had a significant association with age. For the age group 15-45 years, quinolones maintained 90% of susceptibility, except for nalidixic acid, whose susceptibility rate was 78% (Table 1).

**Table 1:** Antimicrobial susceptibility of *E. coli* isolated from women according to age range.

	*< 15*		15-45		46-59		≥ 60		*p*-value*
	*n*	%	n	%	N	%	n	%	
Amikacin	347	100	2130	99.6	1113	99.9	1769	99.5	0.255
Gentamicin	341	98.3	2037	95.2	1059	95.1	1629	91.7	<0.001
Amoxicillin-clavulanic acid	305	87.9	1901	88.9	982	88.2	1518	85.4	0.011
Ampicillin	190	54.8	1203	56.2	601	53.9	924	52.0	0.068
Piperacillin-tazobactan	338	97.4	2091	97.8	1084	97.3	1703	95.8	0.005
Cephalothin	186	53.6	1155	54.0	574	51.5	855	48.1	0.003
Cefepime	339	97.2	2079	97.2	1064	95.5	1635	92	<0.001
Ceftriaxone	341	98.3	2079	97.2	1062	95.3	1625	91.4	<0.001
Cefuroxime	330	95.1	2009	93.9	1020	91.6	1500	84.4	<0.001
Nalidixic acid	279	80.4	1684	78.7	810	72.7	1096	61.7	<0.001
Norfloxacin	330	95.1	1941	90.7	941	84.5	1306	73.5	<0.001
Ciprofloxacin	329	94.8	1938	90.6	940	84.4	1303	73.3	<0.001
Ertapenem	347	100	2135	99.8	1106	99.3	1770	99.6	0.087
Meropenem	347	100	2136	99.9	1108	99.5	1767	99.4	0.059
Nitrofurantoin	334	96.3	2072	96.9	1082	97.1	1698	95.6	0.079
SMX /TMP	255	73.5	1573	73.5	781	70.1	1200	67.5	<0.001
ESBL	07	2.0	54	2.5	47	4.2	147	8.3	<0.001
TOTAL	347	100	2139	100	1114	100	1777	100	

⁎ p valor qui square test or Exact Fisher Test. Variables with significant association: gentamicin, amoxicillin-clavulanic, piperacillin tazobactan, cephalothin, cefepime, ceftriaxone, cefuroxime, nalidixic acid, norfloxacin, ciprofloxacin, SMX/TMP.

The level of antimicrobial resistance to the quinolones tested in this study (nalidixic acid, ciprofloxacin, and norfloxacin) was 15.5%. The susceptibility profile to other first-line antimicrobials used in the treatment of UTIs decreased dramatically, especially to cephalothin, ampicillin and TMP-SMX, which presented only 40% susceptibility in these isolates. The only first-line antimicrobial agent that maintained a rate of susceptibility greater than 90% for these isolates was nitrofurantoin, regardless of age. The presence of ESBL-producing isolates was not significantly associated with age when the isolate was resistant to the quinolones used in our study (Table 2).

**Table 2:** Antimticrobial Susceptibility of samples resistant to three quinolones according to age range.

	*< 15*		15-45		46-59		≥ 60		Valor de p*
	*n*	%	n	%	n	%	n	%	
Amikacin	17	100	146	99.5	166	100	338	99.6	<0.001
Gentamicin	17	100	140	72.9	135	81.3	350	75.9	0.031
Amoxicillin-clavulanic	15	88.2	146	76	130	78.3	338	73.3	0.339
Ampicillin	02	11.8	32	16.7	46	27.7	89	19.3	0.041
Piperacillin-tazobactan	15	88.2	178	92.7	158	95.2	417	90.5	0.246
Cephalothin	05	29.4	62	32.3	58	34.9	127	27.5	0.294
Cefepime	13	76.5	154	80.2	140	84.3	359	77.9	0.349
Ceftriaxone	13	76.5	152	79.2	141	84.9	353	76.6	0.158
Cefuroxime	12	70.6	140	72.9	122	73.5	291	63.1	0.024
Ertapenem	17	100	192	100	161	97	456	98.9	0.076
Meropenem	17	100	192	100	164	98.8	457	99.1	0.414
Nitrofurantoin	16	94.1	184	95.8	159	95.8	426	92.4	0.254
TMP/SMX	07	41.2	81	42.2	78	47	200	43.4	0.809
ESBL	05	29.4	37	19.3	24	14.5	105	22.8	0.102
TOTAL	17	100	192	100	166	100	461	100	

⁎ *p*-value chi-square and Fisher's Exact test. Variables with significant association (*p* < 0.05): Amikacin, gentamicin, cefuroxime, ertapenem.

## Discussion

Our cross-sectional study shows the antimicrobial susceptibility profile of uropathogens of urine cultures collected from women who attended the Basic Health and Emergency Units in Londrina, Southern Brazil. Londrina has 537,377 inhabitants, and is located at 23°18′36“S51°09′46”O.

*E. coli* was the most common pathogen isolated. Furthermore, women aged 15-45 years had the greatest number of positive urine cultures, as observed by other authors.^2^^,^^9^, ^10^^-^^11^

In this study, men were excluded from analysis because they had complicated UTIs, which was not the scope of this work, as also performed by Dubbs et al.^12^ In a study carried out in Curitiba, Brazil, with outpatients who received care at the public health system, Reu et al.^13^ also reported that the lowest frequencies of *E.* coli, as causative agent of UTIs, were found between pregnant women, men, and boys. These data suggest that although *E. coli* was the most common uropathogen, its distribution may vary according to sex and patient physiological status, being less common in men.

Resistance to TMP-SMX was greater than 30% in this study, regardless of age. Specifically, 32.5% of isolates from patients aged 60 years and over showed resistance to this antimicrobial agent. According to Gupta et al.,^7^ TMP-SMX is recommended as the first-line treatment for uncomplicated UTIs, but only when resistance rates to this antibiotic do not exceed 20%.

It is known that antimicrobial resistance varies geographically, including within a country, as shown by Cunha et al.^6^ who reported a resistance rate of 50.6% to TMP-SMX in a Northeastern Brazilian city, while in India, in 2013, the resistance rate to this antibiotic was 52%, increasing up to 59.6% in 2017.^14^ However, in a study carried out in the USA, Yamaji et al.^15^ showed that frequencies of resistance to TMP-SMX in uropathogenic *Escherichia coli* (UPEC) isolates obtained from outpatients with UTI symptoms in 1999–2000 and in 2016–2017 had not increased significantly over the studied period (resistance increased slightly from 16.9% to 17.1%). Studies in which resistance to TMP-SMX is greater than 35% suggest the replacement and/or withdrawal of this antibiotic from first-line treatment of uncomplicated UTIs.^16^ Currently, guidelines have recommended the use of fosfomycin–trometamol and pivmecillinam as first- and second-line treatments for these infections, respectively.^17^

In this study, resistance to quinolones surpassed 10% in isolates from women aged ≥ 46 years, whereas the overall resistance level to quinolones was 15.5%. The association of quinolone resistance with older age had also been observed in the literature.

Quinolones are the most frequently selected antimicrobials for treating uncomplicated UTIs in many countries.^18^^,^^19^ Risk factors associated with resistance to this antimicrobial class include patients older than 60 years of age, presence of obstructive uropathy, recurrent UTI history, as well as the use of quinolones in the past three months.^19^^,^^20^^,^^21^

Despite FDA warnings about the use of quinolones in 2016,^22^ the rate of prescriptions of these antibiotics has not changed over years, and their inappropriate use was more frequent in the treatment of uncomplicated UTIs. Thus, the overuse and side-effects of quinolones must be incorporated into the clinical decision regarding antimicrobial treatment of all infections, such as upper respiratory tract infection, uncomplicated UTIs, and abdominal infections.^23^^,^^24^

Conversely, resistance to nitrofurantoin was very low in our study, less than 5% in all age groups, including the age group > 60 years, even among those isolates resistant to quinolones (7.6%). The same pattern was observed in a study conducted in Rio Grande do Norte, Brazil, in which 6.6% of *E. coli* was resistant to nitrofurantoin (*n* = 653).^6^ Likewise, a retrospective analysis performed by Sanchez et al.^25^ showed that, in the United States, nitrofurantoin retains a high level of antibiotic activity against urinary *E. coli* isolates. Nevertheless, the resistance levels to nitrofurantoin in India and Mexico are among the highest reported worldwide: 3% and 12.7%, respectively.^14^^,^^26^ These results show that nitrofurantoin remains the treatment of choice for uncomplicated UTIs, although it should not be used for the treatment of pyelonephritis, since its concentration in the renal parenchyma is too low.^27^

In this study, fosfomycin was tested for all quinolone-resistant isolates, showing high susceptibility. Similarly, in a study performed in India with 7,295 isolates obtained from patients with uncomplicated UTI, fosfomycin and nitrofurantoin displayed the greatest susceptibility levels.^28^ Other countries in Europe and in the USA also reported high rates of susceptibility to Fosfomycin.^29^^,^^30^ These results highlight the use of fosfomycin as the antibiotic of first choice in the treatment of UTIs.^8^ In Brazil, fosfomycin is an expensive antimicrobial agent, and unlike TPM-SMX, nitrofurantoin, norfloxacin, and ciprofloxacin, it is not available to patients in the public health system. In this scenario, exposure to fosfomycin is a fundamental risk factor which can lead to the selection of resistant *E. coli* isolates.

Among beta-lactams used for uncomplicated UTIs, amoxicillin/clavulanic acid displayed low levels of resistance regardless of age: 12.1%, 11.1%, 11.8% and 14.6%, for age groups < 15, 15-45, 46-59 and ≥ 60 years, respectively. While in Belgium, Germany, and Spain, levels of resistance to cefuroxime (second-generation cephalosporin) were 5.5%, 12.8%, and 16.6%, respectively (30), in this study cefuroxime showed a low resistance rate, similar to reports in the literature, and thus could be a treatment option for community-acquired UTIs in our region.

ESBL-positive isolates were more frequent the older the women, being more common in women over 60. The frequency of ESBL-producing isolates in the present study was 4.7%, which was lower than the 7.6% found by Abreu et al. in Northeast^31^ and 7.1% found by Gonçalves et al. in Central-Western Brazil.^32^ However, the rates were lower in the Southern (0.4%) and South 1.5% of the country.^31^, ^32^, ^33^^-^^34^

ESBL prevalence varies all over the world. A study carried out in Pakistan showed a prevalence of ESBL-production in 33% of *E. coli* isolated from community-acquired UTIs.^35^ Still, in Southern France, approximately 4% of *E. coli* isolates from community-acquired UTIs are ESBL producers.^36^ Prevalence of ESBL might change over time, as shown by Northwestern Memorial Hospital (Chicago, USA), where the percentage of ESBL-producing E. coli among community-onset urine isolates increased from 0.21% in 2003 to 2.99% in 2008, that is, a 14-fold increase within that period. Moreover, it was reported that CTX-M–producing *E. coli* accounted for the majority of ESBLs producers in that hospital.^37^

Our study has a few limitations. First, our data may not represent the real antimicrobial susceptibility profile of bacteria causing uncomplicated UTIs, because urine culture is not recommended at the first episode of uncomplicated UTI. Second, empirical treatment is based on a positive test strip (leukocytes+ or nitrites +) and clinical signs and symptoms. Information concerning patients’ clinical history, prior use of antimicrobials, recurrence of UTI and comorbidities was available. Third, fosfomycin was tested in isolates resistant to the three quinolones used in this study, but not in all isolates, since fosfomycin is expensive and unlike TMP-SMX, ciprofloxacin, norfloxacin, cephalothin, amoxicillin and nitrofurantoin, it is not offered free of charge by Public Health System.

In conclusion, our data show that TMP-SMX should not be considered as an option for first-line treatment of community-acquired UTIs in our region. Conversely, since nitrofurantoin and fosfomycin displayed the lowest resistance levels, they can be chosen as empirical antimicrobial treatment of uncomplicated UTIs. As antimicrobial resistance to quinolones increases with age, the treatment in older women should always be based on urine culture results. In addition, since resistance to the three quinolones tested in this work was 15.5%, empirical treatment for pyelonephritis should be avoided. Finally, stewardship is necessary for rational antimicrobial prescribing, in an attempt to decrease the selective pressure of resistance in our environment, as well as hospital costs related to hospitalization and patient deaths.

## Conflicts of interest

The authors declare no conflicts of interest.
